# Supplementary material for: Hyperloss from coherent spatial-mode mixing in quantum-correlated networks
Source: Nat Commun. 2026 Jul 25;17:7270. doi: 10.1038/s41467-026-75899-5 (PMC13401599; doi:10.1038/s41467-026-75899-5)
Supplement: Supplementary file 1 — Supplementary Information [file 41467_2026_75899_MOESM1_ESM.pdf]

Supplementary material for “Hyperloss from coherent spatial-mode mixing in quantum-correlated networks”

Stephan Grebien[1†], Julian Gurs[1†], Roman Schnabel[1], Mikhail Korobko[1*]

[1]Institut für Quantenphysik and Zentrum für Optische Quantentechnologien, Universität Hamburg, Luruper Chaussee 149, 22761 Hamburg, Germany.

*Corresponding author(s). E-mail(s): mikhail.korobko@uni-hamburg.de;
†These authors contributed equally to this work.

Abstract

This supplementary material provides additional details on the experimental setup, data evaluation, squeezing results, calibration procedures and theoretical analysis used in the study of hyperloss in quantum-correlated networks.

Supplementary Note 1 - Experimental setup and phase evolution of hyperloss

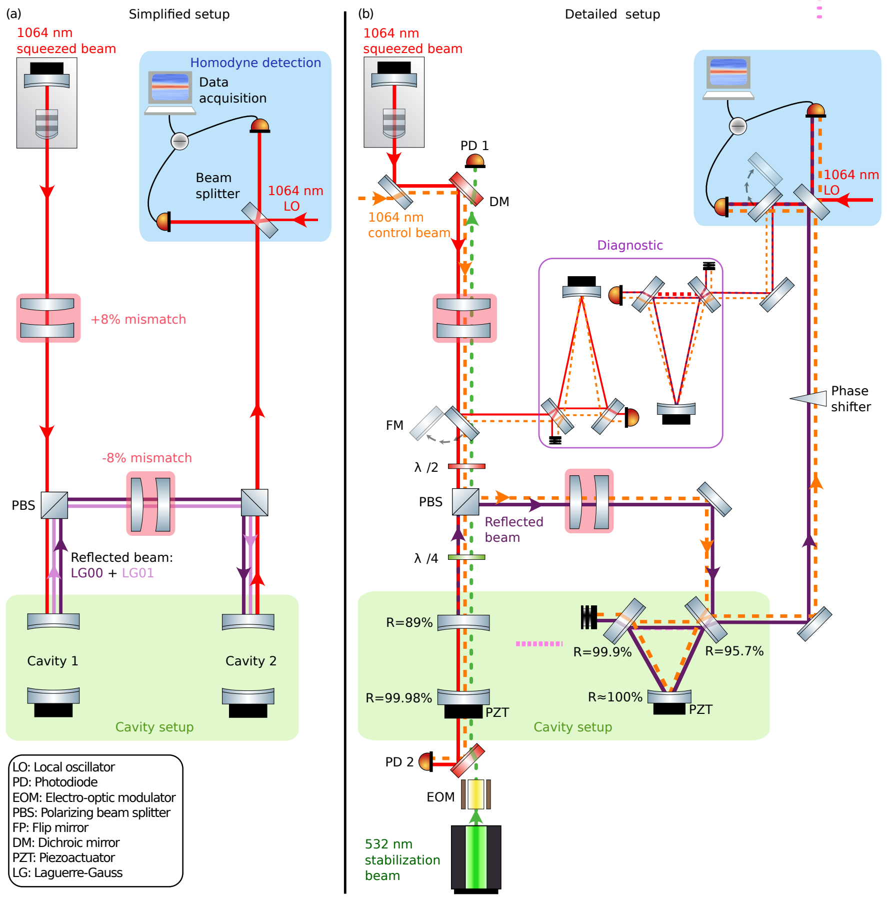

Supplementary Figure 1: Left: A simplified setup highlighting the main components of the experiment: the introduced mismatch of ±8 % (red background), the cavity setup (green background), and the homodyne detection system (blue background). The experiment uses Lagueere-Gauss modes (LG_{00} and LG_{01}) to realize the fundamental and higher-order spatial modes. The first set of lenses creates +8 % mismatch at a cavity 1, and the second set of lenses compensates it at cavity 2, resulting in pure LG_{00} mode at the homodyne detector. Right: A detailed setup, additionally including auxiliary components such as a control beam to assist in aligning the squeezed beam via the diagnostic setup and a stabilization beam for cavity 1.

Supplementary Table 1: Optical properties of the used cavities.

	Squeezing Cavity	Cavity 1	Cavity 2
Wavelength (nm)	1064	1064	1064
Finesse	66	53.8	79
Free spectral range (GHz)	5.28	33.3	1.3
Linewidth (MHz)	80	618	16.5

Supplementary Figure 1 shows a schematic illustration of the experiment, with the left side depicting a simplified setup and the right side a more detailed configuration. Here, we focus on the detailed case. Starting with the 1064 nm squeezed beam (red), this originates from a non-linear cavity that generates 532 nm light using a Mephisto Nd:YAG laser at 1064 nm via the non-linear process of second harmonic generation. The 532 nm are used to drive a parametric down-conversion below threshold to produce a squeezed vacuum state. The properties of the squeezing cavity are summarized in Tab. 1.

Due to the nature of this setup, the squeezed beam is accompanied by a weak control beam. This weak beam was used to match the squeezed beam to a diagnostic cavity, allowing a strong control beam (orange) to be introduced and matched to the squeezed beam by aligning it to the same diagnostic cavity. As a result, a strong control beam with the same trajectory as the squeezed beam is obtained, which is necessary for aligning the squeezed beam to cavity 1 and cavity 2. Photodiode 2 was used to match the strong control field to the first cavity (properties listed in Tab. 1) passing a lab-made optical isolator, which is composed of a $\frac{\lambda}{2}$-waveplate, $\frac{\lambda}{4}$-waveplate, and a polarizing beam splitter.

Cavity 1 can be length-stabilized using a Pound-Drever-Hall locking scheme with a 532 nm stabilization beam and photodiode 1. Five different stabilization points were identified, visualized as gray-highlighted 532 nm resonance peaks (see Supplementary Figure 2), each corresponding to the respective differential phase response. The phase between the 532 nm and 1064 nm resonance peaks is defined by Φ and is set to 0 for double resonance, corresponding to the hyperloss case.

Next, the control beam and the mixture of fundamental and higher-order modes (purple, alias reflected beam) are sent to cavity 2 (details in Tab. 1), which was permanent length tuned. In the final step, the control beam is overlapped with a 1064 nm local oscillator at a 50:50 beam splitter, aided by a diagnostic mode cleaner. This procedure results in a fully aligned setup. However, intentionally, a mode mismatch of $+8\,\%$ was introduced in the off-resonance case at cavity 1, leading to the generation of higher-order modes, which are then compensated at cavity 2.

We measured hyperloss with a home-made homodyne detector consisting of two output ports: one for the DC signal and one for the AC signal (high-pass filtered DC signal). Both outputs were recorded using an acquisition card made by Advantech, model PCIe-1840L, with a sampling rate of 80 MHz and an anti-aliasing filter. The DC port was used to record the beat between the weak control beam and the local oscillator, while the AC port was used to record the squeezed light field. It should be noted that the DC port was low-pass filtered to remove noise from the squeezed state, which was not phase-synchronized to the DC reference.

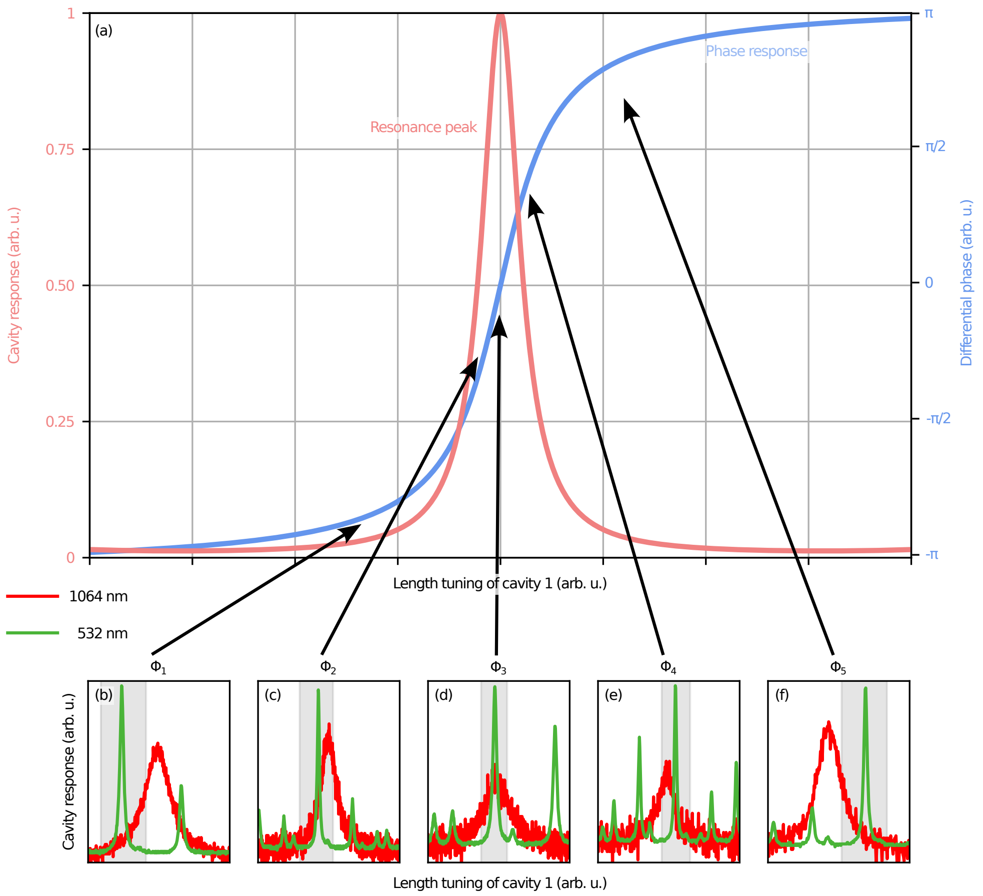

Supplementary Figure 2: Top: Phase response of cavity 1's resonance peak at different length stabilization points. Bottom: Resonance peaks of 1064 nm (monitored at photodiode 2) and 532 nm (monitored at photodiode 1) for cavity 1. Different modes of 532 nm were optimized to achieve various differential phases between the fundamental and higher-order modes in response to the 1064 nm resonance peak.

A more detailed evaluation can be seen in Supplemenatry Figure 3. Here, an evolution of the effect is shown based on the five possible stabilization points.

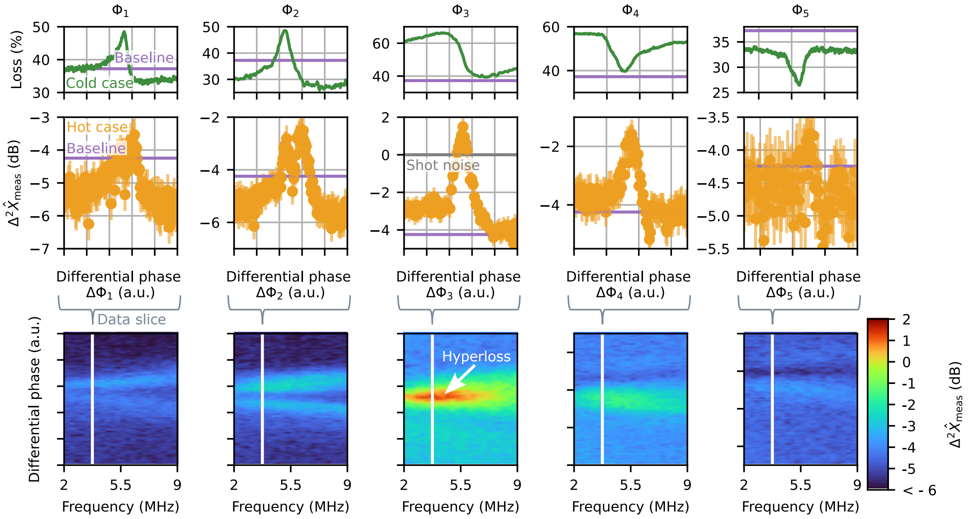

Supplementary Figure 3: The figure illustrates the evolution of hyperloss for different stabilization points of cavity 1, leading to varying differential phases as a function of these phases. The top row displays the classical losses, which are derived from the beat between the strong control beam and the local oscillator. The second row presents the hot case. Both the transition to a thermal like state and coherent restoration are visible, in comparison to the baseline, which accounts for the mismatch as a direct loss. Column (Φ_3) depicts the regime where measurements are above shot noise, reaching a notably thermal like state. Column (Φ_2) shows the maximal recovery relative to the baseline (off-resonance) case. Importantly, the cold and hot cases exhibit significantly different behaviors, confirming the quantum nature of the effect. Error bars indicate the noise level estimation uncertainty, as described in the Supplementary Note 2. The third row shows the experimental spectrogram, illustrating the squeezing as a function of measurement frequency and differential phase. White lines in the third row indicate the frequency (3.75 MHz) at which the slices in the top two rows were taken. Dotted lines serve as guidelines.

Supplementary Note 2 - Data evaluation

We begin the data evaluation by dividing the measurements into time bins i, each consisting of 51.2 million samples. First, the amplitude of the interference was analyzed by examining the maximum and minimum values within each time bin of the DC signal and calculating their difference. The output power $P_{\text{meas}}[i]$ was then estimated by squaring this value. This power estimation was subsequently repeated for the reference measurement (i.e., both cavities are off resonance) to obtain an estimate of $P_{\text{in}}[i]$. Finally, the loss was calculated using Eq. 1 in the Main text.

To estimate the squeezed light field, we first segmented the data from the AC port into the same time bins as the DC signal. Subsequently, a spectrogram was computed using

the `scipy.signal.spectrogram` function of the Python package `scipy` (version 1.6.2), running on Python (version 3.8.8). In detail, the used parameters for the function are listed in Tab. 2. After computing the spectrogram, it was smoothed using a moving average filter (MAF) with a window size of 100 to reduce statistical noise in the power estimation. The squeezing and anti-squeezing values are estimated as the minimum and maximum of their respective time and frequency bins.

Supplementary Table 2: Parameters used for the `scipy.signal.spectrogram` function.

Parameter	Value
Sampling frequency (`fs`)	80,000,000
Segment length (`nperseg`)	256
Overlap between segments (`noverlap`)	128
Window type (`window`)	Tukey, shape parameter 0.25

Impact of the moving average filter size

The size of the moving average filter strongly influences our results, leading either to an underestimation or overestimation of the effect (or, more specifically, the measured squeezing), as shown in Supplementary Figure 4. We identify three regions: first, from sizes 10 to 100, there is a strong overestimation of the values. Second, between sizes 100 and 1000, the estimates remain relatively stable, up to sizes larger than 1000, where the values are strongly underestimated.

We face two counteracting effects that influence our squeezing estimates. First, overestimation due to a statistical uncertainty in our power estimation from the spectrogram measurement. Second, underestimation caused by averaging over a large phase angle. A larger MAF size reduces the error in our power estimation but increases the average phase angle and vice versa. To find a good compromise between these two effects, we select a filter size that corresponds to the value given by the classical losses.

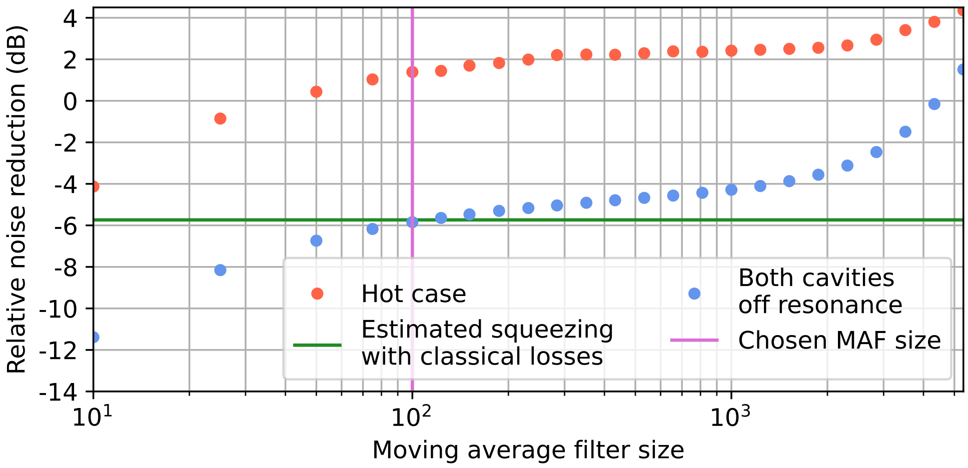

Supplementary Figure 4: To obtain a squeezing estimate for each evaluation, we take the trace at 3.125 MHz and average the squeezing values over time bins 200 to 300. The green line shows the squeezing estimate from our calibration losses of (26.3 ± 3.4) %, while the pink line shows the later chosen MAF size. The squeezing for the reference was estimated by taking the minimum for each time-bin while both cavities were off-resonance and average over them, blue dots. For the chosen MAF size of 100 our squeezing estimate from this procedure is below the green line, ensuring that we underestimate the effect. To compare the impact of the MAF size on the effect we plot it in in red, alias hot case. Both lines exhibit similar behavior until, for large MAF sizes of 2000, the effect of averaging over a large phase angle begins to dominate.

An evolution of hyperloss as a spectrum for various moving average filter sizes can be seen in Supplementary Figure 5. Three regions can be identified: Below approximately 100, we tend to overestimate the measured squeezing, compared to the expected true value. This overestimation gradually decreases, leading to an underestimation of squeezing at an MAF of around 1000. Above approximately 2800, additional artifacts appear. These are caused by the large phase angle over which we average, combined with phase drift, since the squeezing was not phase-stabilized to the local oscillator.

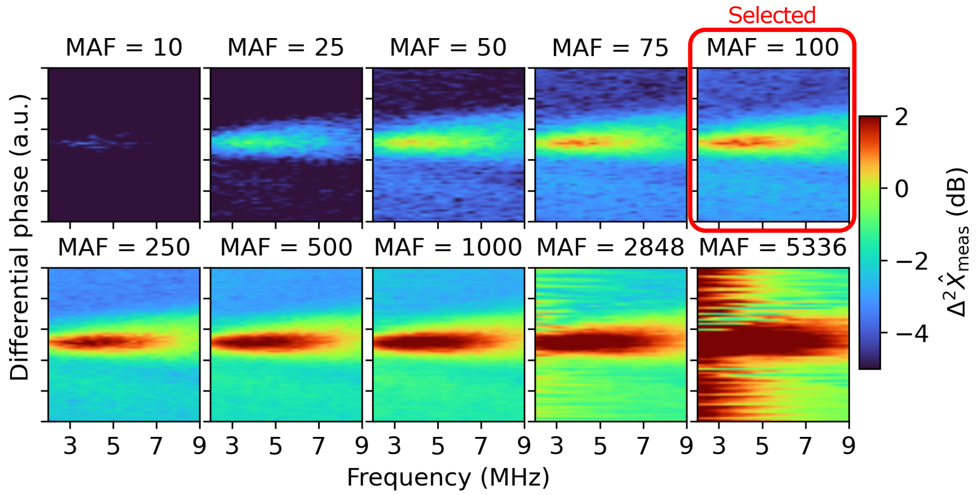

Supplementary Figure 5: Visualization of various moving average filter size as a squeezing spectrogram. More detailed information in the text.

Supplementary Note 3 - Squeezing results

In this section, we aim to demonstrate the effect of hyperloss in the ”typical” squeezing diagrams. For this, cavity 1 is set to the differential phase Φ_3. Supplementary Figure 6 is divided into three panels: at the bottom row, spectrograms of squeezing (left side) and anti-squeezing (right side), both within a measurement frequency range of 2.5 MHz to 9.25 MHz at different positions of cavity 2. A slice at 3.75 MHz is shown in the top panel. The evolution of the measured state from quantum to thermal and vice versa is shown as cavity 2 length is tuned. Quantum states are distinguished by anti-squeezing (light red dots, top half) and squeezing (blue dots, bottom half), with the thermal state highlighted in yellow. A clear transfer from approximately (-2.5 $\pm$ 0.5) dB to approximately (1.4 $\pm$ 0.5) dB and back can be observed, with red dots indicating the transferred squeezing data. Meanwhile, anti-squeezing increases throughout the full length tuning. The uncertainty is dominated by the evaluation error, as discussed above.

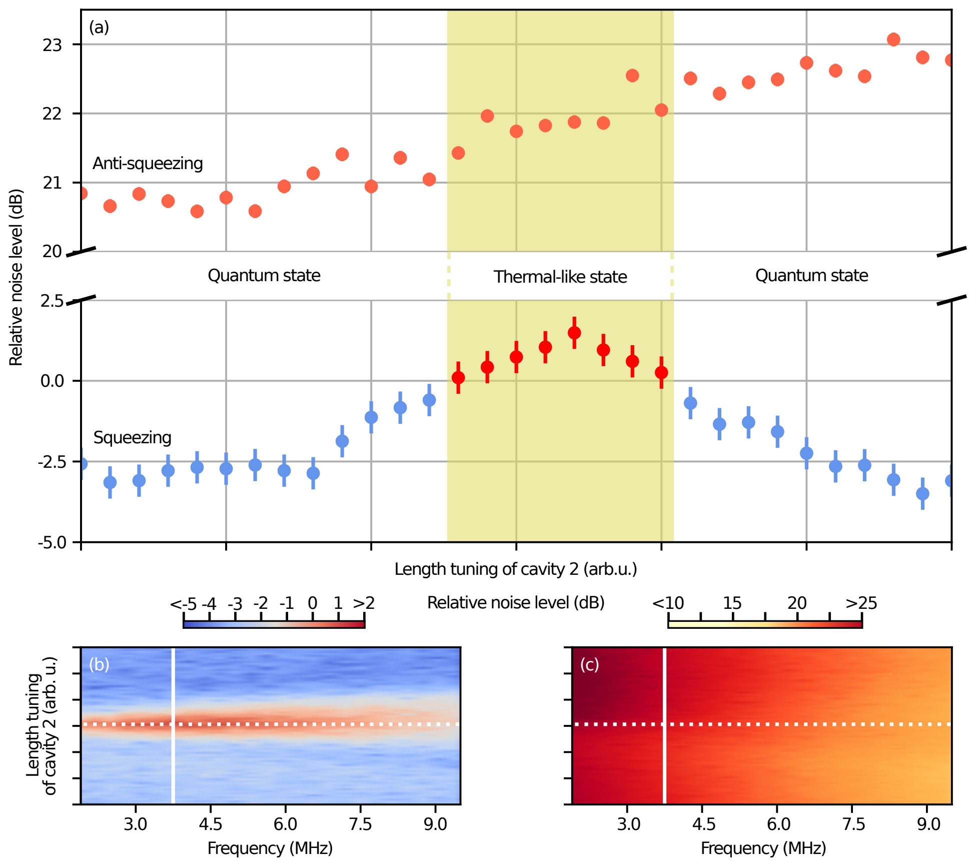

Supplementary Figure 6: Top image: The minimal (light blue) and maximal (light red) variance of the noise at a measurement frequency of 3.75 MHz as a function of the differential phase between the FM and the HOM. Squeezed quadrature transitions to a thermal-like state when the hyperloss effects occurs. The hyperloss regime where all quadratures exceed the shot noise is highlighted in yellow. Data points where the squeezing directly evolves into a thermal-like state are marked in red. Error bars indicate the noise level estimation uncertainty, as described in the Supplementary Note 2. Bottom: Spectrogram of squeezing (left) and anti-squeezing (right). The vertical white line indicates the frequency of 3.75 MHz, corresponding to the top image, while the dashed horizontal white line marks the tuning of cavity 2 with the strongest observed hyperloss effect.

An increase in anti-squeezing and a decrease in squeezing directly indicate non-classical behavior. In Supplementary Figure 7 (right) the effect of classical loss on squeezed

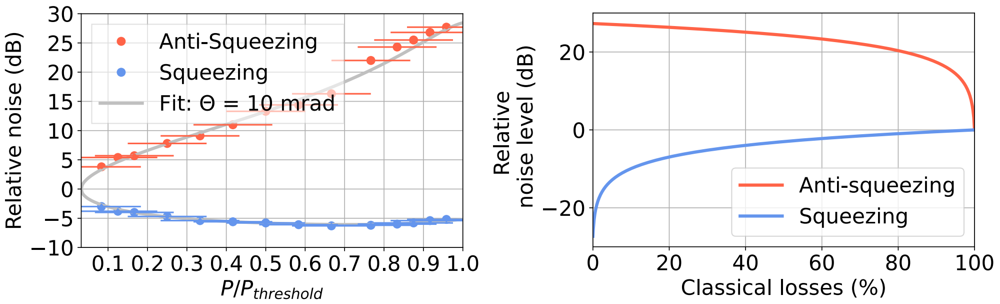

Supplementary Figure 7: Left: Phase noise estimation of our squeezed state with both cavities in an off-resonance case, displaying squeezing in blue and anti-squeezing in red. The uncertainty is defined by the measurement uncertainty of thepower meter. Right: Simulation the behavior of classical losses on squeezed states.

light was simulated, using the analytical equation [1]:

$$\Delta^2 \hat{X}_{0,\pi/2}(P) = 1 \pm \eta \frac{4\sqrt{\frac{P}{P_{\mathrm{thr}}}}}{\left(1 \mp \sqrt{\frac{P}{P_{\mathrm{thr}}}}\right)^2 + 4\left(\frac{\nu}{\Gamma}\right)^2}. \tag{1}$$

With η the detection efficiency, the threshold power P_{thr}, the measurement frequency ν and the decay rate of the cavity Γ. For the sake of completeness, we also checked the phase noise of the source of occasion, as shown in Supplementary Figure 7. For this purpose, both cavities were set to off-resonance, and squeezing (blue dots) and anti-squeezing (red dots) were measured at different pump powers relative to the threshold power $P_{\mathrm{thr}} = (120 \pm 12)\,\mathrm{mW}$ of the squeezer resonator. The uncertainty is given as $10\,\%$ based on the power meter OPHIR model Nova II. Our results are verified by theory using the analytical equation [1]:

$$\Delta^2 \hat{X}^{\mathrm{m}}_{0,\pi/2}(\Theta) = \Delta^2 \hat{X}_{0,\pi/2} \cos(\Theta)^2 + \Delta^2 \hat{X}_{\pi/2,0} \sin(\Theta)^2. \tag{2}$$

With $\Delta^2 \hat{X}_{0,\pi/2}$ representing the measured squeezing and anti-squeezing, and Θ the phase noise, measured as $(10 \pm 1)\,\mathrm{mrad}$, dominated by the uncertainty of the powermeter. All this confirms that the effect of hyperloss is real.

Supplementary Note 4 - Calibration

We have estimated our classical loss contribution; the results can be seen in Tab. 3. Most components are specified by the manufacturer, e.g., the general propagation loss towards the homodyne detection. This is relatively minor due to the use of high-quality mirrors ($R \geq 99\,\%$) and is provided by the manufacturer LASEROPTIK. The same applies to all other common components such as Faraday isolators, lenses, photodiodes, and dichroic beam splitters (DBSs).

We defined a set of optics, labeled as Set 1 in the table, which could not be distinguished individually. This set was measured as a single component using a power meter, and the individual losses were estimated accordingly. The accuracy of these estimation is limited by the measurement precision of the power meter OPHIR model Nova II with 10 %.

The escape efficiency of the squeezed light source is given by $\frac{T}{T+L}$, where T is the transmissivity of the coupling mirror and L is the sum of all round-trip losses. These include scattering and absorption losses, as well as residual transmission through the crystal's imperfectly reflecting rear face. The manufacturer provided the transmissivity of the coupling mirror and the crystal characteristics, which were then used to determine the escape efficiency and convert it to the loss ($\eta - 1$).

The overlap between the local oscillator and squeezed light field was analyzed with an

Supplementary Table 3: Overview of all optical Losses

**Source**	**Loss (%)**
Propagation loss	1
Faraday isolator	2.3
Lenses	0.3
DBS front squeezer	0.1
DBS front cavity one	0.5
Waveplate	0.2
Set 1 measured:<br>*PBS, λ/4-waveplate, Mirror, Cavity 1*	= 16.3 ± 10.0
Intracavity on-resonance	0.3
Intracavity off-resonance	1.1
Escape efficiency as loss of the squeezer	1 ± 0.1
Quantum efficiency of photodiodes	0.5
Visibility	2 ± 0.2
Phase noise equivalent	(0 to 4) ± 0.1
Estimated losses	(26.3 to 30.3) ± 10.4

interference measurement. For this purpose we used the well aligned control, instead of the squeezed beam. Quantification was done by using the visibility V given by the formula

$$V = \frac{I_{\text{max}} - I_{\text{min}}}{I_{\text{max}} - I_{\text{min}}}. \tag{3}$$

with I_{max} representing the maximum of the interference fringe and I_{min} the minimum. Note that the estimated classical loss value cannot be directly compared with the

squeezing values, as the conditions were slightly different for each measurement, specifically in terms of phase matching temperature, pump power, and data evaluation. We always aimed to obtain the maximum squeezing and anti-squeezing values. Estimating the introduced loss for cavity 1 is not trivial, since photodiode 2 was sensitive to the individual modes differently depending on the beam position.

Supplementary Note 5 - Simplified theoretical model

In this section we consider the simplified theoretical model that allows to approximately compute the observed effects and perform analytical analysis. We start by using the Mach-Zender model in Fig. 2 of the Main text, where there are two modes mixed on a cavity or a beam-splitter. We use the matrix formalism and express the state as large correlation matrix for the FM (denoted by f) and HOM (denoted by h), for two quadratures x, y. Using the two-mode formalism [2] we write quadrature squeezing directly, defining $S_{mn,xx} = \Delta^2 \hat{X}_0(\Omega), S_{mn,xy} = \Delta^2 \hat{X}_{\pi/2}(\Omega), S_{mn,xy} = S_{yx} = \Delta^2 \hat{X}_{\pi/4}(\Omega)$, where the index mn denotes the variance (when $m = n$) or cross-correlation (when $m \neq n$) between the FM and the HOM. The initial state in this notation is given by:

$$S_{\text{init}} = \left[\begin{array}{cc|cc} S_{ff,xx} & S_{ff,xy} & S_{fh,xx} & S_{fh,xy} \\ S_{ff,yx} & S_{ff,xx} & S_{fh,yx} & S_{fh,yy} \\ \hline S_{hf,xx} & S_{hf,xy} & S_{hh,xx} & S_{hh,xy} \\ S_{hf,yx} & S_{hf,yy} & S_{hh,yx} & S_{hh,xx} \end{array}\right] = \left[\begin{array}{cc|cc} e^{2r_a} & 0 & 0 & 0 \\ 0 & e^{-2r_s} & 0 & 0 \\ \hline 0 & 0 & 1 & 0 \\ 0 & 0 & 0 & 1 \end{array}\right], \tag{4}$$

where $r_{a,s}$ are the squeeze factors of the anti-squeezed and squeezed quadratures, with difference accounting for the losses experienced by the state before injection in the network. In the initial state, the FM is squeezed, while the HOM is in the vacuum state, and there are no cross-correlations between them. We separate the action of the coupling interface (a beam splitter or a cavity) into two parts: the mixing of the modes and the mixing of the quadratures. Mode mixing can be simply defined by the rotation matrix for the non-diagonal elements in each quadrant for relatively small mismatches [3, 4] for interface 1 and 2:

$$\mathbb{T}_{1,2} = \begin{bmatrix} \cos k_{1,2} & 0 & -\sin k_{1,2} & 0 \\ 0 & \cos k_{1,2} & 0 & -\sin k_{1,2} \\ \sin k_{1,2} & 0 & \cos k_{1,2} & 0 \\ 0 & \sin k_{1,2} & 0 & \cos k_{1,2} \end{bmatrix}, \tag{5}$$

where we defined $k_{1,2}$ as the amplitude coupling coefficient between the modes for interface 1 and 2, correspondingly. We note that this coupling model is valid for small mismatches, where only the coupling to a single HOM is significant. For larger mismatches, multiple HOMs will be excited, and the model needs to be extended accordingly. For simplicity, we assume that only the HOM acquires quadrature rotation (in the full model this simplification is not applied), due to the coupling at mixing interface or the Gouy phase.

$$\mathbb{O}_{1,2} = \begin{bmatrix} 1 & 0 & 0 & 0 \\ 0 & 1 & 0 & 0 \\ 0 & 0 & \cos \phi_{1,2} & -\sin \phi_{1,2} \\ 0 & 0 & \sin \phi_{1,2} & \cos \phi_{1,2} \end{bmatrix} \tag{6}$$

The input-output relation for the field quadratures x, y for modes f and h are given by:

$$\begin{pmatrix} x_{f,\text{meas}} \\ y_{f,\text{meas}} \\ x_{h,\text{meas}} \\ y_{h,\text{meas}} \end{pmatrix} = \mathbb{O}_2\mathbb{T}_1\mathbb{O}_1\mathbb{T}_1 \begin{pmatrix} x_{f,\text{in}} \\ y_{f,\text{in}} \\ x_{h,\text{in}} \\ y_{h,\text{in}} \end{pmatrix} \tag{7}$$

The output noise is then given by:

$$S_{\text{meas}} = \begin{pmatrix} x_{f,\text{meas}} \\ y_{f,\text{meas}} \\ x_{h,\text{meas}} \\ y_{h,\text{meas}} \end{pmatrix} \begin{pmatrix} x_{f,\text{meas}} & y_{f,\text{meas}} & x_{h,\text{meas}} & y_{h,\text{meas}} \end{pmatrix} = \mathbb{O}_2\mathbb{T}_1\mathbb{O}_1\mathbb{T}_1 S_{\text{init}} \left(\mathbb{O}_2\mathbb{T}_1\mathbb{O}_1\mathbb{T}_1\right)^T \tag{8}$$

We are interested in one quadrature (e.g. phase) of the fundamental mode, therefore, we only take element (2,2) of the resulting matrix:

$$\begin{aligned} S_{y,\text{meas}} = S_{\text{meas}}[[2,2]] = (\cos k_2 \sin k_1 + \cos k_1 \cos\phi_1 \sin k_2)^2 + \\ e^{-2r_s} (\cos k_1 \cos k_2 - \cos\phi_1 \sin k_1 \sin k_2)^2 + \cos^2 k_1 \sin^2 k_2 \sin^2\phi_1 + \\ e^{2r_a} \sin^2 k_1 \sin^2 k_2 \sin^2\phi_1. \end{aligned} \tag{9}$$

We highlight three special cases $\phi = \{0, \pi/2, \pi\}$, corresponding to Fig. 2, assuming equal mismatch at two interfaces, $k_1 = k_2$:

$$S_{y,\text{meas}}(\phi_1 = \pi) = e^{-2r_s}, \tag{10}$$

$$S_{y,\text{meas}}(\phi_1 = 0) = \sin^2 2k_1 + e^{-2r_s} \cos^2 2k_1, \tag{11}$$

$$S_{y,\text{meas}}(\phi_1 = \pi/2) = e^{-2r_s} \cos^4 k_1 + 2\cos^2 k_1 \sin^2 k_1 + e^{2r_a} \sin^4 k_1. \tag{12}$$

In the first case, squeezing is perfectly coherently restored, despite two mismatches — this is the restoration effect. In the second case, squeezing is still present, although is significantly suppressed: e.g. for the mismatch of 8 %, $S_{y,\text{meas}} \approx \sqrt{0.08}(1 + 2.5e^{-2r_s})$. Finally, the third case is most dramatic, where anti-squeezing couples directly into the squeezed quadrature. For example, for the mismatch of 8 %:

$$S_{y,\text{meas}}(\phi_1 = \pi/2, k_1 = \sqrt{0.08}) \approx 0.01 e^{2r_a}, \tag{13}$$

where we assumed that anti-squeezing is strong enough, $e^{2r_a} \gg 1$. In this case, we observe complete decoherence of the quantum state for any input squeezing stronger than 20 dB, for which $e^{2r_a} = 100$. Higher input squeezing or stronger mode coupling leads to hyperloss – a significantly mixed state.

We also consider the case of a weak coupling, where $k_{1,2} \ll 1$. We expand sine and cosine up to the second order in k_1, k_2, and up to the fourth order for the term proportional to e^{2r_s}, since it can be significant if anti-squeezing is strong enough:

$$S_{y,\text{meas}} \approx e^{-2r_s}(1 - \lambda_{\text{smm}}) + \lambda_{\text{smm}} + T, \tag{14}$$

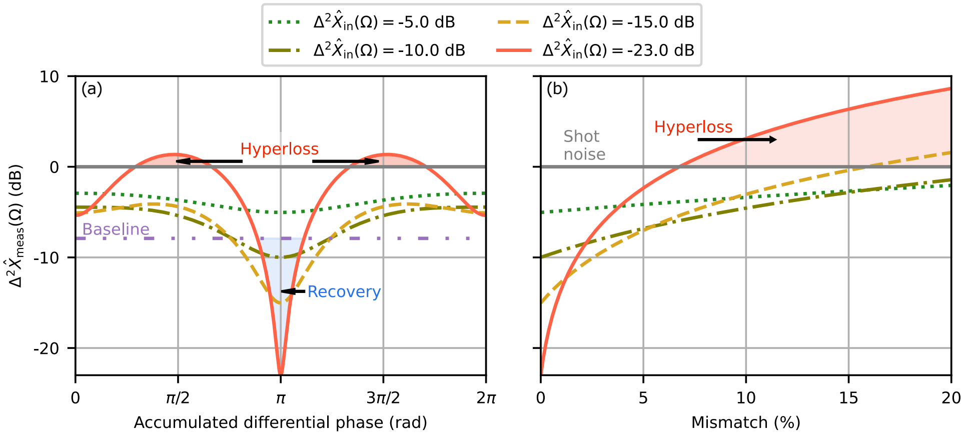

Supplementary Figure 8: The effect of hot SMM on a squeezed state. Left: Noise reduction below the shot noise level as a function of the phase difference between the FM and HOM, for different levels of initial squeezing. The effect of hyperloss is visible for $\pi/2$ phase difference, and the effect of recovery – for π phase difference. The mode mismatch is equal to 8 % on two nodes. Right: Noise reduction below the shot noise level as a function of the mode mismatch, for different levels of initial squeezing and $\phi = \pi/2$. The higher the initial squeezing is, the less tolerant the system is to the mismatch, entering the hyperloss regime for moderate amounts of mismatch.

$$\lambda_{\text{smm}} = k_1^2 + k_2^2 + 2k_1k_2\cos\phi_1, \tag{15}$$

$$T = k_1^2 k_2^2 e^{2r_a}\sin^2\phi_1, \tag{16}$$

where we introduced the effective loss λ_{smm} and the effective normalized noise temperature T. This equation is leads to Eq.3 in the main text. We again highlight three special cases:

$$\phi_1 = \pi: \qquad \lambda_{\text{smm}} = (k_1 - k_2)^2, \quad T = 0, \tag{17}$$

$$\phi_1 = 0: \qquad \lambda_{\text{smm}} = (k_1 + k_2)^2, \quad T = 0, \tag{18}$$

$$\phi_1 = \pi/2: \qquad \lambda_{\text{smm}} = k_1^2 + k_2^2, \quad T = k_1^2 k_2^2 e^{2r_a}. \tag{19}$$

In the last case, the anti-squeezing is coupling into squeezed field, leading to severe decoherence and creation of a mixed thermal-like state. Supplementary Figure 8 shows the dependence of the measured squeezing on the phase difference and the mode mismatch and highlights both hyperloss and recovery effects.

The same treatment could be applied to derive the cold SMM effect. In this case the initial state is the classical amplitude, with only one non-zero element in the

correlation matrix:

$$S_{\text{init,class}} = \left[\begin{array}{cc|cc} P_{\text{in}} & 0 & 0 & 0 \\ 0 & 0 & 0 & 0 \\ \hline 0 & 0 & 0 & 0 \\ 0 & 0 & 0 & 0 \end{array}\right], \tag{20}$$

resulting in a ”cold” SMM effect for small mismatches:

$$P_{\text{meas}} = P_{\text{in}} \left(1 - k_1^2 - k_2^2 - 2k_1k_2 \cos\phi_1\right). \tag{21}$$

Network analysis

We extend the simplified model from the previous section to analyze the effect of the mismatch on one path in the large-scale quantum network. We consider a series of mixing components, each defined by a mode-mixing matrix $\mathbb{T}$ and a quadrature-rotation matrix $\mathbb{O}$. Each mode then acquires a phase shift and loss, defined by the propagation matrix $\mathbb{H}$. The overall transformation of the quadratures is then given by the series of multiplications of these matrices:

$$\begin{pmatrix} x_{f,\mathrm{meas}} \\ y_{f,\mathrm{meas}} \\ x_{h,\mathrm{meas}} \\ y_{h,\mathrm{meas}} \end{pmatrix} = \prod_{i=1}^{N} \mathbb{O}_i \mathbb{T}_i \begin{pmatrix} x_{f,\mathrm{in}} \\ y_{f,\mathrm{in}} \\ x_{h,\mathrm{in}} \\ y_{h,\mathrm{in}} \end{pmatrix}, \tag{22}$$

where N is the number of mixing components in the path. The output noise is then given by:

$$S_{\mathrm{meas}} = \prod_{i=1}^{N} \mathbb{O}_i \mathbb{T}_i S_{\mathrm{init}} \left(\prod_{i=1}^{N} \mathbb{O}_i \mathbb{T}_i \right)^T . \tag{23}$$

While the realistic experimental setups may include different coupling strength for each component, and a variety of overall phase shifts, we can provide the envelope of the expected effect by assuming equal coupling strength $k_i = k$ and equal phase shifts $\phi_i = \phi$. The results of this consideration is discussed in the Results 2.3 section of the main text.

Supplementary Note 6 - Exact numerical modeling

In order to construct the full numerical model for our experiment, we follow the general approach in Ref. [5]. Unlike the previous section, where we considered the evolution of the quadratures, here we will explicitly describe the evolution of a sideband field, and compute noise spectral densities from that [2]. Such approach allows treating upper and lower entangled sidebands of squeezed field independently, to include phase rotations and losses on each of them individually. This is also done for both FM and HOM.

The exact treatment of the mode conversion is rather complex: for an arbitrary mismatch, FM may scatter into multiple HOMs, and depending on the specific type of mismatch (e.g. wavefront curvature mismatch or optical axis tilt mismatch), coupling matrices differ. This should be the subject of the independent study. Here we limit ourselves to the case of a small enough value of mismatch ($< 10\,\%$), such that the FM scatters into the lowest order HOM. In this case, is possible to assign an exact scattering coefficient depending on the specific cavity geometry and alignment [4], but we will consider a generic value of mismatch Υ, which experimentally quantifies the power scattered into the HOM. With this assumption, we define the coupling between the two modes as the rotation of the basis,

$$\mathbb{U}_{1,2} = e^{i\psi_{1,2}} \begin{pmatrix} \sqrt{1-\Upsilon_{1,2}} & -e^{i\phi_{1,2}}\sqrt{\Upsilon_{1,2}} \\ e^{-i\phi_{1,2}}\sqrt{\Upsilon_{1,2}} & \sqrt{1-\Upsilon_{1,2},} \end{pmatrix}, \tag{24}$$

where we defined the coupling strength $\Upsilon_{1,2}$, the relative coupling phase $\phi_{1,2}$ and the overall phase difference $\psi_{1,2}$ (although this phase is experimentally controlled to zero, and we will set it accordingly in the computation).

Next, we define the reflection coefficients for each mode in each cavity:

$$R_1 = -\frac{(\gamma_1 - \lambda_1) - i(\Omega - \delta_1)}{(\gamma_1 + \lambda_1) + i(\Omega - \delta_1)}, \tag{25}$$

$$R_2 = -\frac{(\gamma_2 - \lambda_2) - i(\Omega - \delta_2)}{(\gamma_2 + \lambda_2) + i(\Omega - \delta_2)}, \tag{26}$$

$$R_{1\ \mathrm{hom}} = 1, \tag{27}$$

$$R_{2\ \mathrm{hom}} = 1, \tag{28}$$

$$\mathbb{H}_{1,2} = \begin{pmatrix} R_{1,2} & 0 \\ 0 & R_{1,2\ \mathrm{hom}} \end{pmatrix}, \tag{29}$$

where $\gamma_{1,2}$ are the optical linewidths, $\lambda_{1,2}$ are the losses in the cavity (in units of frequency), Ω is the sideband frequency and $\delta_{1,2}$ is the detuning from cavity resonance. We assume that the cavities are not resonant for the high-order mode, and it gets directly reflected.

Finally, we define the relative Gouy phase shift upon propagation such, that it only applies to the HOM:

$$\mathbb{G} = \begin{pmatrix} 1 & 0 \\ 0 & e^{i\psi_g} \end{pmatrix}. \tag{30}$$

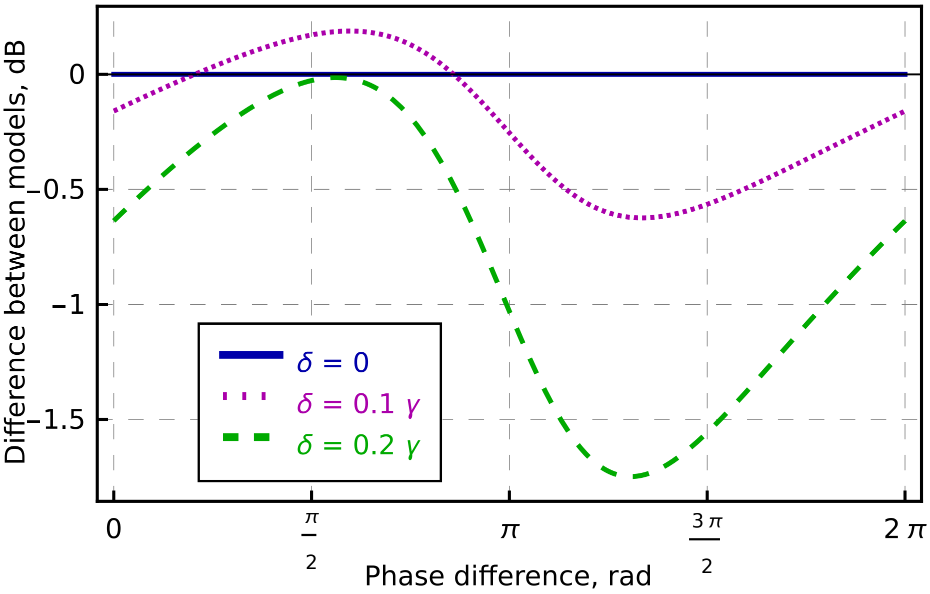

Supplementary Figure 9: Difference between the simplified and full model for the noise degradation due to hot SMM, as a function of the relative phase between the FM and HOM. For the on-resonance case (solid blue) the simplified model perfectly matches the full model. The detuned cases (green and magenta) show more complex behaviors, with frequency-dependent loss.

Then the propagation of the fundamental mode can be defined by the series of interactions (we choose only the first element in the resulting matrix since we are interested in the fundamental mode only):

$$h = \begin{pmatrix} 1 & 0 \end{pmatrix} \mathbb{U}_{2h}\mathbb{H}_2\mathbb{U}_2\mathbb{G}\mathbb{U}_{1h}\mathbb{H}_1\mathbb{U}_1 \begin{pmatrix} 1 \\ 0 \end{pmatrix}, \tag{31}$$

$$h_m = h\Big|_{\Omega\to-\Omega}. \tag{32}$$

This propagation matrix allows to define three terms, corresponding dephasing, propagation efficiency and effective coupling between two quadratures (including hot SMM effects):

$$\Theta = \frac{\arg(h) + \arg(h_m)}{2}, \tag{33}$$

$$\eta_r = \frac{|h|^2 + |h_m|^2}{2}, \tag{34}$$

$$\Xi = \frac{(|h| - |h_m|)^2}{4\eta_r}. \tag{35}$$

This gives the overall coupling between the two quadratures:

$$N_p = (1 - \Xi)e^{2r} + \Xi e^{-2r}, \tag{36}$$
$$N_m = (1 - \Xi)e^{-2r} + \Xi e^{2r}, \tag{37}$$
$$N = N_m \cos^2(\zeta + \Theta) + N_p \sin^2(\zeta + \Theta), \tag{38}$$

where we defined the homodyne angle ζ. Finally, in order to compute the measured noise spectral density, we include propagation efficiency between two cavities η_p, injection efficiency η_i and detection efficiency η_d:

$$S_{\text{meas}}(\Omega) = \eta_i\eta_r\eta_p\eta_d N + (1 - \eta_i\eta_r\eta_p\eta_d). \tag{39}$$

This measured noise includes several effects:

- direct propagation losses, including intra-cavity loss;
- frequency-dependent squeezing, where for a detuned cavity squeeze phase rotates with frequency;
- dephasing due to the imbalanced loss between the two sidebands in a detuned cavity;
- hot SMM effects, including hyperloss and recovery effects.

We note that in this general treatment, hot SMM contributes in a complex way to the sensitivity, including non-trivial frequency dependence.

While it is not possible to extract a comprehensible analytical expression from these matrix equations, we can compare it to the results of the simple treatment presented in the previous section. For this, we assume the cavities to be identical and no losses to be present except for the mode mismatch. If the cavities are on resonance, the computed noise should be identical for both treatments for Fourier frequency componens close to the cavity resonance, $\Omega \ll \gamma_{1,2}$. In Supplementary Figure 9 we show the difference between the two models as a function of phase between the modes, and also compare it to the case when in the full treatment the cavities are detuned (the simple treatment does not cover this case). As expected, when the cavities are tuned, hot SMM is identical in two computations, confirming the consistency of our models.

This model is used to compute the theoretical curves in Fig. 3 in the main text, using the actual experimental parameters, including cavity linewidths, detunings, and losses, as defined in the experimental description in the previous sections.

References

[1] Mehmet, M. *et al.* Squeezed light at 1550 nm with a quantum noise reduction of 12.3 db. *Opt. Express* **19**, 25763–25772 (2011). URL https://opg.optica.org/oe/abstract.cfm?URI=oe-19-25-25763.

[2] Danilishin, S. L. & Khalili, F. Y. Quantum measurement theory in gravitational-wave detectors. *Living Reviews in Relativity* **15** (2012).

[3] Töyrä, D. *et al.* Multi-spatial-mode effects in squeezed-light-enhanced interferometric gravitational wave detectors. *Phys. Rev. D* **96**, 022006 (2017). URL https://link.aps.org/doi/10.1103/PhysRevD.96.022006.

[4] Goodwin-Jones, A. W. *et al.* Transverse mode control in quantum enhanced interferometers: a review and recommendations for a new generation. *Optica* **11**, 273–290 (2024). URL https://opg.optica.org/optica/abstract.cfm?URI=optica-11-2-273.

[5] McCuller, L. *et al.* LIGO's quantum response to squeezed states. *Physical Review D* **104**, 062006. URL https://link.aps.org/doi/10.1103/PhysRevD.104.062006.
