# Supplementary material for: Hyperloss from coherent spatial-mode mixing in quantum-correlated networks
Source: Nat Commun. 2026 Jul 25;17:7270. doi: 10.1038/s41467-026-75899-5 (PMC13401599; doi:10.1038/s41467-026-75899-5)
Supplement: Supplementary file 2 — Transparent Peer Review File [file 41467_2026_75899_MOESM2_ESM.pdf]

nature portfolio

Peer Review File

Hyperloss from coherent spatial-mode mixing in quantum-correlated networks

Corresponding Author: Dr Mikhail Korobko

This file contains all reviewer reports in order by version, followed by all author rebuttals in order by version.

Version 0:

Reviewer comments:

Reviewer #1

(Remarks to the Author)
The article shows novel results regarding the impact of coherent spatial mode-mixing from mode mismatch which are especially relevant for any application in the field of optics which depends on quantum-correlations as is the case of gravitational-wave detectors. The novel concept of hyperloss, which is rigorously demonstrated experimentally, might become crucial in the design of quantum-correlated networks and future gravitational-wave detectors which make use of squeezed-states of light. The experiment is described in detail and the theoretical concepts and calculations are shown clearly and are in agreement with the experimental results.

Below I provide minor comments and typo corrections and afterwards some comments or questions aimed at improving the clarity of the manuscript.

Minor Comments/typos:
-Repeated in "in in" in the 6th line of the abstract.
-Rpeated from "from from photonic quantum processes" in the last paragraph of the abstract.
-Fig. 3. Perhaps I have missed it, but I think in the caption of Fig. 3. there should be a mention that the simulated results were obtained with the numerical model described in the supplement.
-Page 8, CV acronym not introduced. Is it continuous variable ? It's only used once so maybe just write continuous variable, otherwise makes the text unnecessarily more opaque.
-Page 16 "squeezing is still present, although is significantly suppressed" – (suggestion) to "squeezing is still present, although significantly suppressed."
-Page 10 of the supplement – I think the acronym DBS is never introduced, which I imagine stands for dichroic beam splitter.

Questions/comments to clarify:
- Perhaps it is my own lack of understanding, but I thought the physical meaning of the auxiliary and main modes used to describe the mode mismatch mechanism around Fig. 1. could be better explained. It isn't entirely clear to me. Can the auxiliary mode be the vacuum mode at the beam splitter, for example ? In the cavity, the main mode seems to be the cavity eigenmode and the auxiliary is the input mode to the cavity ?
- Is Fig. 7 made assuming equal squeezing and anti-squeezing (no injection losses) ? According to the Mathematica code provided it seems it is. Perhaps it would be beneficial to say this explicitly in the caption or when mentioning Fig. 7, as the right panel might be confusing when comparing with the results of the experiment of Fig. 3. or 6. Because in the experiment hyperloss is achieved with less than 6 dB squeezing and 8% mode mismatch, while in the right panel of Fig. 7 the hyperloss is not even crossed for 5dB squeezing. I understand this discrepancy is due to the injection losses in the experiment and the high amount of anti-squeezing with respect squeezing ra > rs, but perhaps it would be useful to make this clear in the discussion. It wasn't clear to me at first.
- General question, perhaps to be addressed in the conclusions or in a couple of places mentioned below. I feel the impact of other HOMs could be explained or explored a bit further, even if the article and experimental realization focus only on the LG01 from small amounts of mode mismatch. In particular, see below:
-- At the beginning of page 9, can careful phase design account for hyperloss from various HOMs as they accrue different Gouy phases ? Or might other solutions be required in this case, such as the HOM squeezing mentioned elsewhere in the article ?
-- In the experiment described in this article, other HOMs are accounted at 2%. Perhaps I'm wrong, but I would imagine that

hyperloss for even a small HOM could lower significantly the achievable squeezing. However, the theoretical model with a single HOM seems to match well the experimental results. Is it because the HOMs are spread out and their multiple phase contributions somehow cancel ?
- One final question, perhaps not needed to address in the article unless relevant: If I understand correctly the results shown here are for squeezing in the MHz range. Does the picture change in any way or would there be any other considerations in the case of audio-band squeezing as used in gravitational-wave detectors ?

Ricardo Cabrita

(Remarks on code availability)
Although I am not experienced with Mathematica, the code seems to run well and reproduce the relevant figures shown in the article. Even though there is no README file the comments in the mathematica notebook are helpful to understand which parameters were used for plotting.

Reviewer #2

(Remarks to the Author)
This manuscript presents an experimental investigation into the "hyperloss" phenomenon. In this effect, mode mismatching in a single-mode squeezed vacuum degrades the squeezing level more severely than conventional vacuum loss. Hyperloss occurs when the anti-squeezing component in higher-order modes (HOMs) couples into the target measurement mode. This phenomenon can lead to critical errors in implementing various quantum information processes that require high squeezing and modal interference, such as quantum sensing, continuous-variable quantum computing, and quantum communication. Therefore, mitigating hyperloss will aid future progress in this field.

In this manuscript, the authors demonstrated hyperloss by interfering the fundamental mode (LG00) and an HOM (LG01) with a lens set, then manipulating their relative phase via cavity detuning. They also mitigated hyperloss and recovered the initial squeezing level by actively controlling the HOM phase. This paper presents highly interesting results, and I generally support its publication in Nature Communications. However, the manuscript requires considerable clarification on the following points before it is fully suitable for publication:
1. In Equation (1), which describes the cold-phase-dependent SMM loss, as well as in the subsequent expression for the phase-independent loss, the expansion is truncated at the k^2 term. It should be explicitly stated that this approximation is only valid in the small-k regime.
2. The authors extend their demonstrated method to a simulated multimode interferometric system. They assume identical transmittance and phase rotation across all interferometers. However, the manuscript does not clearly explain how phase compensation was applied to correct hyperloss in these simulations. Unlike the single-mode scenario, a multimode system theoretically needs phase adjustment at every interferometer where phase rotation occurs. In a practical setup, implementing full phase control is highly challenging. Realistically, compensation might only be feasible at interferometers closest to the measurement stage. A discussion on whether effective hyperloss mitigation remains possible under such real-world limitations would greatly benefit the manuscript.

(Remarks on code availability)

Version 1:

Reviewer comments:

Reviewer #1

(Remarks to the Author)
All the comments have been addressed perfectly and clarified. Thank you to the authors for the clear and detailed answers.

Unless I misunderstand, I believe there is a small typo in the caption of Fig. 8, where it reads: "assuming a pure initial state, i.e. no equal squeeze and anti-squeeze values." it should read ".., i.e. equal squeeze and anti-squeeze values", without the "no".

(Remarks on code availability)

Reviewer #2

(Remarks to the Author)
The authors revised their manuscript addressing the points raised in my review. In particular, I can agree with the discussion in the current version about the simulation of the interferometric system. They clearly stated that the discussion in chapter 2.3 is not a practical compensation scheme, but the effect of hyperloss accumulation on the multiple interferometric system. In addition, the modified interpretations were explained in a well-structured manner to support the paper’s discussion.

I recommend this paper for publication in Nature Communications.

(Remarks on code availability)

Open Access This Peer Review File is licensed under a Creative Commons Attribution 4.0 International License, which permits use, sharing, adaptation, distribution and reproduction in any medium or format, as long as you give appropriate credit to the original author(s) and the source, provide a link to the Creative Commons license, and indicate if changes were made.
In cases where reviewers are anonymous, credit should be given to 'Anonymous Referee' and the source.
The images or other third party material in this Peer Review File are included in the article's Creative Commons license, unless indicated otherwise in a credit line to the material. If material is not included in the article's Creative Commons license and your intended use is not permitted by statutory regulation or exceeds the permitted use, you will need to obtain permission directly from the copyright holder.
To view a copy of this license, visit https://creativecommons.org/licenses/by/4.0/

We thank the Reviewers for their valuable input on our manuscript. We have carefully addressed the comments one-by-one. We believe it has improved the clarity of the manuscript and it is now ready for publication. Below we list our response point-by-point and also attach the marked version of the manuscript.

Reviewer #1 (Remarks to the Author):

> The article shows novel results regarding the impact of coherent spatial mode-mixing from mode mismatch which are especially relevant for any application in the field of optics which depends on quantum-correlations as is the case of gravitational-wave detectors. The novel concept of hyperloss, which is rigorously demonstrated experimentally, might become crucial in the design of quantum-correlated networks and future gravitational-wave detectors which make use of squeezed-states of light. The experiment is described in detail and the theoretical concepts and calculations are shown clearly and are in agreement with the experimental results.

We thank the Reviewer for careful reading of our paper, for the positive evaluation of our work and for highlighting its importance for the future of quantum technology. We respond to the comments one-by-one. The comments are indented and in blue, our response is in black.

Minor Comments/typos:

> -Repeated in “in in” in the 6th line of the abstract.

Corrected.

> -Rpeated from "from from photonic quantum processes" in the last paragraph of the abstract.

Corrected.

> -Fig. 3. Perhaps I have missed it, but I think in the caption of Fig. 3. there should be a mention that the simulated results were obtained with the numerical model described in the supplement.

The reference to the Supplementary is added.

> -Page 8, CV acronym not introduced. Is it continuous variable ? It’s only used once so maybe just write continuous variable, otherwise makes the text unnecessarily more opaque.

The acronym is no longer used.

> -Page 16 “squeezing is still present, although is significantly suppressed” – (suggestion) to "squeezing is still present, although significantly suppressed."

We accepted the suggested version.

> -Page 10 of the supplement – I think the acronym DBS is never introduced, which I imagine stands for dichroic beam splitter.

We added a definition.

Questions/comments to clarify:

> - Perhaps it is my own lack of understanding, but I thought the physical meaning of the auxiliary and main modes used to describe the mode mismatch mechanism around Fig. 1. could be better explained. It isn't entirely clear to me. Can the auxiliary mode be the vacuum mode at the beam splitter, for example ? In the cavity, the main mode seems to be the cavity eigenmode and the auxiliary is the input mode to the cavity ?

We thank the Reviewer for highlighting the lack of clarity on this point. The auxiliary mode must be a well-defined mode in the basis of the fundamental mode. Vacuum field does not have a selected basis (since all possible modes are populated by the vacuum field). To avoid any ambiguity, we now renamed the auxiliary mode to the mismatched mode in Fig. 1 and updated the caption to make this more clear.

> - Is Fig. 7 made assuming equal squeezing and anti-squeezing (no injection losses) ? According to the Mathematica code provided it seems it is. Perhaps it would be beneficial to say this explicitly in the caption or when mentioning Fig. 7, as the right panel might be confusing when comparing with the results of the experiment of Fig. 3. or 6. Because in the experiment hyperloss is achieved with less than 6 dB squeezing and 8% mode mismatch, while in the right panel of Fig. 7 the hyperloss is not even crossed for 5dB squeezing. I understand this discrepancy is due to the injection losses in the experiment and the high amount of anti-squeezing with respect squeezing ra > rs, but perhaps it would be useful to make this clear in the discussion. It wasn't clear to me at first.

We thank the Reviewer for highlighting this. Indeed, the assumption is that the injected state is pure. Also, every conventional loss contributes to the overall limitation on the detected squeezing. Therefore, even though we start with 23 dB of squeezing/anti-squeezing in the experiment, it quickly deteriorates to the level that is observed (about 5.8 dB). We added the clarifying statement in the caption of Fig. 7 and the surrounding discussion.

> - General question, perhaps to be addressed in the conclusions or in a couple of places mentioned below. I feel the impact of other HOMs could be explained or explored a bit further, even if the article and experimental realization focus only on the LG01 from small amounts of mode mismatch. In particular, see below:
>
> -- At the beginning of page 9, can careful phase design account for hyperloss from various HOMs as they accrue different Gouy phases ? Or might other solutions be required in this case, such as the HOM squeezing mentioned elsewhere in the article ?

There are two main ways to impact the differential phase: directly by changing the Gouy phase (e.g. by free space propagation or with focusing elements) and by introducing mode-selective phase shifts (e.g. by using additional filter cavities or using spatial light modulators). The former approach impacts all HOMs at the same time, thus optimising the phase of one HOM may degrade the phase of the other. The latter approach allows to have more fine-tuned approach to this. However, in practice it depends on the strength of the specific modes and on the available degrees of freedom. In some cases, squeezing the HOM could be a viable solution. We added a clarifying statement that a combination of multiple strategies would likely be required to address the multi-mode coupling.

> -- In the experiment described in this article, other HOMs are accounted at 2%. Perhaps I'm wrong, but I would imagine that hyperloss for even a small HOM could lower significantly the achievable squeezing. However, the theoretical model with a single HOM seems to match well the experimental results. Is it because the HOMs are spread out and their multiple phase contributions somehow cancel ?

We estimated the total impact of all remaining modes to be below 2%, with each individual contribution being significantly smaller than that. Also, most of them would not be coupled coherently: each cavity had its own response and we optimised only for the coherent coupling of the LG01 mode. Therefore, these modes can be treated mostly an incoherent loss source. Even if a small portion of them did couple coherently, this contribution was within the error margins of our parameter set used in a simulation and would not have a significant impact.

We also note that we observe a good qualitative match between the theory and the experiment, but quantitatively there are differences within ~1dB depending on the frequency. We attribute these differences to the uncertainties in the parameters and also possibly the impact of other HOMs.

We added a clarifying statement in the caption of Fig. 3.

> - One final question, perhaps not needed to address in the article unless relevant: If I understand correctly the results shown here are for squeezing in the MHz range. Does the picture change in any way or would there be any other considerations in the case of audio-band squeezing as used in gravitational-wave detectors ?

We do not think there is any conceptual difference between the MHz range presented here and the audioband range.

Reviewer #2

> This manuscript presents an experimental investigation into the "hyperloss" phenomenon. In this effect, mode mismatching in a single-mode squeezed vacuum degrades the squeezing level more severely than conventional vacuum loss. Hyperloss occurs when the anti-squeezing component in higher-order modes (HOMs) couples into the target measurement mode. This phenomenon can lead to critical errors in implementing various quantum information processes that require high squeezing and modal interference, such as quantum sensing, continuous-variable quantum computing, and quantum communication. Therefore, mitigating hyperloss will aid future progress in this field.
>
> In this manuscript, the authors demonstrated hyperloss by interfering the fundamental mode (LG00) and an HOM (LG01) with a lens set, then manipulating their relative phase via cavity detuning. They also mitigated hyperloss and recovered the initial squeezing level by actively controlling the HOM phase. This paper presents highly interesting results, and I generally support its publication in Nature Communications. However, the manuscript requires considerable clarification on the following points before it is fully suitable for publication:

We thank the Reviewer for the positive evaluation of our work and for the useful remarks to the manuscript. We have addressed them in the following.

> 1. In Equation (1), which describes the cold-phase-dependent SMM loss, as well as in the subsequent expression for the phase-independent loss, the expansion is truncated at the k^2 term. It should be explicitly stated that this approximation is only valid in the small-k regime.

We have explicitly stated that after equation 1.

> 2. The authors extend their demonstrated method to a simulated multimode interferometric system. They assume identical transmittance and phase rotation across all interferometers. However, the manuscript does not clearly explain how phase compensation was applied to correct hyperloss in these simulations. Unlike the single-mode scenario, a multimode system theoretically needs phase adjustment at every interferometer where phase rotation occurs. In a practical setup, implementing full phase control is highly challenging. Realistically, compensation might only be feasible at interferometers closest to the measurement stage. A discussion on whether effective hyperloss mitigation remains possible under such real-world limitations would greatly benefit the manuscript.

We thank the Reviewer for this important point. We agree that in a generic multimode network, practical mitigation may only be feasible at a limited number of late-stage interferometric nodes, and that full phase-aware compensation at every mode-mixing location would be challenging. Our intention in Fig. 8 was not to model such a practical compensation scheme, but rather to provide an idealized coherent-limit envelope by assuming identical repeated cells with $k_i = k$ and $\phi_i = \phi$. We have clarified this explicitly in the Network analysis section and now state that this repeated-cell model should be understood as an intuitive illustration of coherent hyperloss accumulation, rather than as a practical compensation prescription for a generic multimode network. We also softened the interpretation of the recovery regime in Fig. 8 by noting that realistic systems with non-identical phases, multiple HOM channels, and unequal coupling strengths are expected to be less recoverable under limited local control. In addition, we now state in the Conclusion that the achievable degree of mitigation will depend on the specific platform, available control points, and application requirements.
